# Consequences of Viral Infection and Cytokine Production During Pregnancy on Brain Development in Offspring

**DOI:** 10.3389/fimmu.2022.816619

**Published:** 2022-04-07

**Authors:** Daniela Elgueta, Paola Murgas, Erick Riquelme, Guang Yang, Gonzalo I. Cancino

**Affiliations:** ^1^Center for Integrative Biology, Facultad de Ciencias, Universidad Mayor, Santiago, Chile; ^2^Escuela de Tecnología Médica, Facultad de Ciencias, Universidad Mayor, Santiago, Chile; ^3^Escuela de Biotecnología, Facultad de Ciencias, Universidad Mayor, Santiago, Chile; ^4^Department of Medical Genetics, Cumming School of Medicine, University of Calgary, Calgary, AB, Canada; ^5^ Department of Biochemistry and Molecular Biology, Cumming School of Medicine, University of Calgary, Calgary, AB, Canada; ^6^Alberta Children’s Hospital Research Institute, Calgary, AB, Canada

**Keywords:** maternal infections, virus, cytokines, neuroinflammation, neurodevelopmental disorders, cortical development, SARS – CoV – 2, zika virus

## Abstract

Infections during pregnancy can seriously damage fetal neurodevelopment by aberrantly activating the maternal immune system, directly impacting fetal neural cells. Increasing evidence suggests that these adverse impacts involve alterations in neural stem cell biology with long-term consequences for offspring, including neurodevelopmental disorders such as autism spectrum disorder, schizophrenia, and cognitive impairment. Here we review how maternal infection with viruses such as Influenza A, Cytomegalovirus, and Zika during pregnancy can affect the brain development of offspring by promoting the release of maternal pro-inflammatory cytokines, triggering neuroinflammation of the fetal brain, and/or directly infecting fetal neural cells. In addition, we review insights into how these infections impact human brain development from studies with animal models and brain organoids. Finally, we discuss how maternal infection with SARS-CoV-2 may have consequences for neurodevelopment of the offspring.

## Introduction

The mother’s health and her environment can strongly influence the brain development of her offspring (1–3). For example, viral infection of the mother during pregnancy has been linked to increased risk of neurodevelopmental defects in the offspring, such as microcephaly, autism spectrum disorder (ASD), epilepsy, and schizophrenia (4–33). These effects on the offspring may be a consequence of the mother’s systemic immune response against infection, which includes production of maternal cytokines that can interfere with fetal neural stem/precursor cells (NPCs) and microglia (34–37). Viruses can also infect the placenta and thereby pass vertically from mother to fetus, entering NPCs, microglia, and neural cells in the developing fetal brain (38, 39).

Viruses and pro-inflammatory cytokines can damage the nervous system by inhibiting the genesis of NPCs, which are self-renewing, multipotent stem cells capable of giving rise to neurons, astrocytes, and oligodendrocytes (40). Normally the number and fate of NPCs are determined by a balance between their maintenance as NPCs and their differentiation and commitment to form parts of neural circuits. However, viruses and cytokines can throw off this balance, resulting in neurodevelopmental disorders or cortical defects (41, 42). These factors can also harm microglia, the immune cells of the brain that monitor against inflammation and try to ensure normal functioning of neurons (43). Although not themselves neurons, microglia play important roles in neural circuits, such as by regulating synaptic activity and controlling the pool of NPCs (44–46).

This review focuses on how viral infection of the mother can trigger defects in fetal neurodevelopment. We examine three viruses whose effect on brain development has been well studied in animal and cellular models: Zika virus (ZIKV), Cytomegalovirus (CMV), and Influenza A virus. Next we explore the specific pathways triggered in the fetal brain by pro-inflammatory cytokines produced by the mother in response to viral infection. Finally, we consider the possibility that the emerging severe acute respiratory syndrome coronavirus 2 (SARS-CoV-2) may have long-term consequences on fetal brain development.

## Maternal Viral Infections During Fetal Neurodevelopment

**Figure 1:**
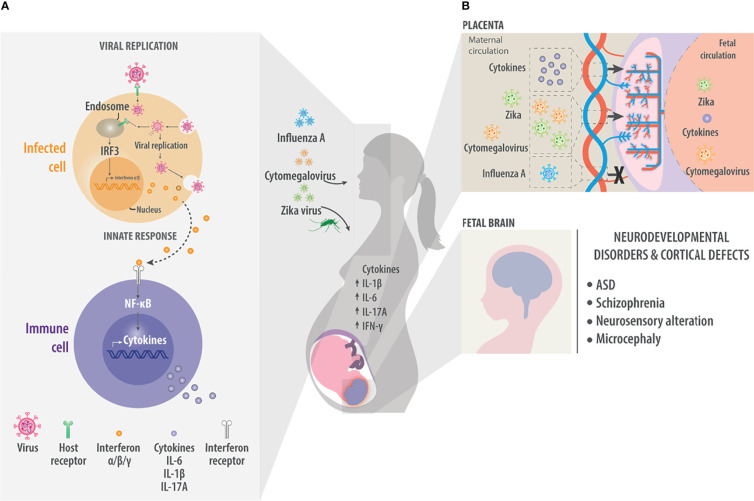
Maternal viral infection. As a result of maternal viral infection, immune responses generate pro-inflammatory cytokines such as IL-1β, IL-6, and IL-17A. These cross the placenta and generate neuroinflammation in the fetal brain. **(A)** During viral infection, molecules on the viral surface are recognized and trigger a cascade of events in the infected cell. Host receptors recognize viral molecules or nucleic acids and initiate a signaling cascade that ends with the nuclear translocation of transcription factors such as interferon regulatory factor 3 (IRF3), which induce the synthesis of type I interferons α and β, which are ultimately released into the extracellular environment. There the interferons are detected by cells of the innate immune system. Innate immune system cells, such as macrophages or dendritic cells, recognize pathogen-associated molecular patterns (PAMPs) and damage-associated molecular patterns (DAMPs). They also respond to cytokines such as interferons to activate signaling pathways *via* NF-κB to generate pro-inflammatory cytokines such as IL-6 and IL-1β. These factors trigger inflammation and recruit more cells to inhibit viral replication or kill infected cells. **(B)** Maternal infection by the Influenza A, Cytomegalovirus or Zika virus generates an increase in maternal pro-inflammatory cytokines that can reach the fetal circulation through the placenta. In the same way, viruses such as Cytomegalovirus or Zika also cross the placenta and directly affect the fetal brain. In the fetal brain, the presence of pro-inflammatory cytokines or viruses can result in neurodevelopmental disorders and defects in the formation of the cerebral cortex, manifesting effects such as ASD, Schizophrenia, neurosensory alteration or microcephaly.

Viruses are microorganisms that require the host's cellular machinery to replicate and then infect new cells or integrate their genetic material into the host genome. To enter host cells, viruses must express diverse molecules such as proteins or carbohydrates on their surface that can be recognized by host cell receptors (47). The innate immune system recognizes these same viral surface molecules, which acts as the first line of defense. When so-called “pattern recognition receptors” on the surface of immune cells or inside the cells recognize pathogen-associated molecular patterns on the virus, the immune cells begin to produce pro-inflammatory cytokines and chemokines by recruiting adapter proteins such as myeloid differentiation primary response 88 (MYD88), TIR-domain-containing adapter-inducing interferon-β (TRIF), and translocating chain-associated membrane protein (TRAM) to initiate a signaling cascade that culminates in the activation of nuclear factor kappa B (NF-κB), mitogen-activated protein kinase (MAPK) and interferon regulatory factors (IRFs). These transcription factors induce the expression of interferon (IFN) α and β as well as pro-inflammatory cytokines such as interleukin (IL)-6, tumor necrosis factor (TNF)-α and IL-1β, which recruit more immune cells to destroy infected cells (48, 49) (**Figure 1A**). The immune cells also induce the adaptive immune system to produce antibodies against the pathogen *via* signaling involving the major histocompatibility complex II, CD40, CD80 and CD86.

Many viruses can have the potential to extend an effect on the fetal brain and trigger pro-inflammatory responses in this way, with potentially serious consequences for the developing brain. Such pathogens include rubella virus (50, 51), herpes simplex virus (52–54) and parvovirus (55). Three viruses that show strong tropism toward neural cells and linked to neurodevelopmental disorders are ZIKV, Influenza A virus and CMV. All three have been associated with ASD, schizophrenia, neurosensory alterations, hydrocephaly, or microcephaly (**Figure 1B**) (4–30).

### Zika Virus

**Figure 2:**
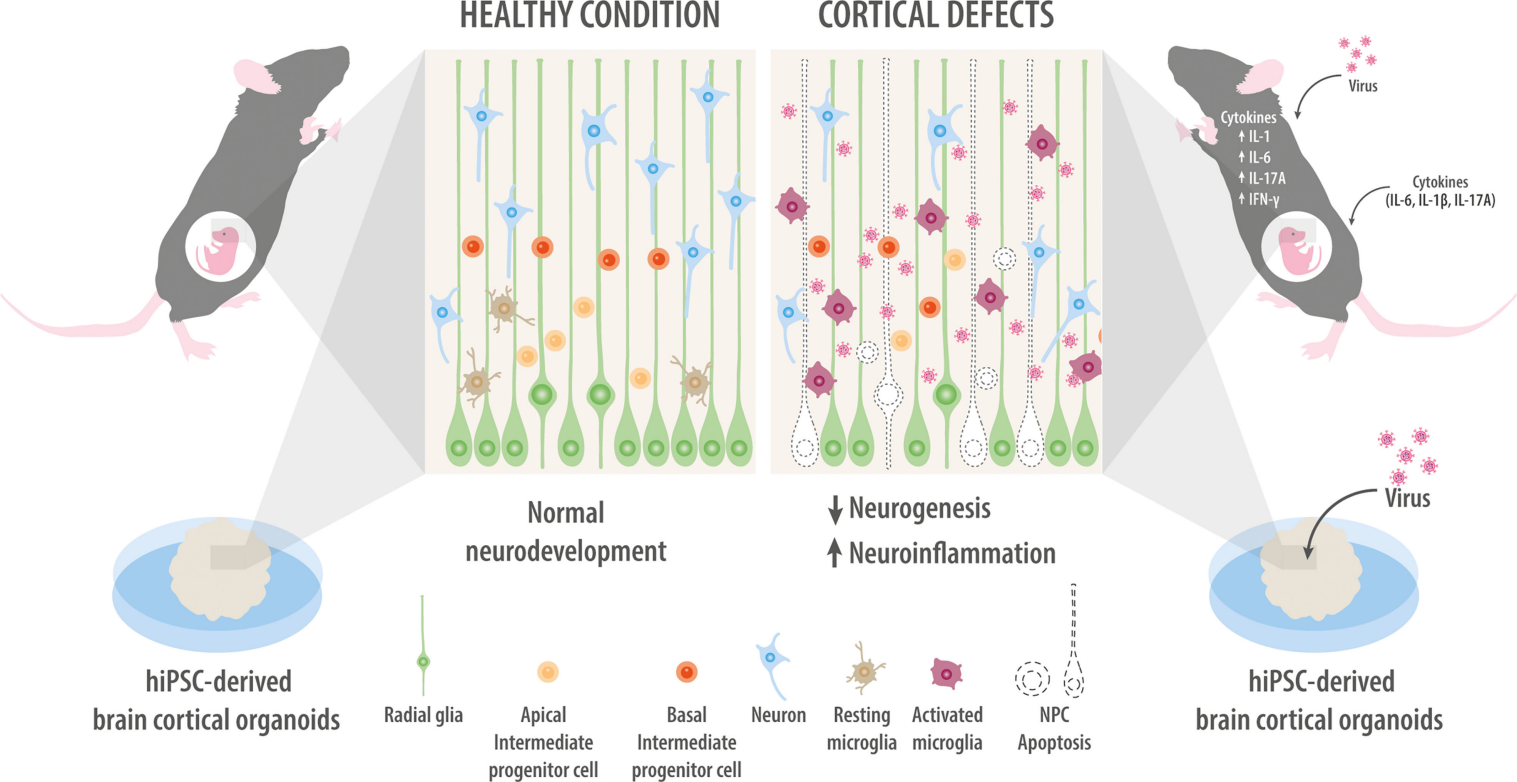
Models of maternal infection. Modeling of maternal infection in mice either by injection of viruses or cytokines (upper right panel). Maternal infection activates microglia and neuroinflammation while inhibiting NPC proliferation and neuronal migration. Infecting brain organoids with Zika virus or Cytomegalovirus inhibits NPC proliferation and promotes their apoptosis, ultimately shrinking the organoid (bottom right panel). For comparison, mock-infected NPCs are intact and show normal organization of the different cortical layers, as well as normal levels of neuronal migration and survival (left panel).

ZIKV is one of the most well-studied viruses that perturb brain development. It is a single-stranded RNA virus, it belongs to the family *Flaviviridae* and genus *Flavivirus* (56). The most evident consequence of ZIKV infection of pregnant mothers is microcephaly in the offspring, and a causal link between the two was implied by the simultaneous presence of the viral genome in cerebrospinal fluid and antibodies in neonates (31–33). Furthermore, animal models in which the virus is injected into the uterus or directly into the fetal brain ventricle have provided important insights into how ZIKV disrupts brain development (**Figure 2**) (57). These models link virus infection with decreased brain volume, disorganization of neuronal layers in the cortex, and apoptosis in the hippocampus and cortex (58), as well as with disruptions of NPC biology that mimic the etiology of several neurodevelopmental disorders (41, 59). In fact, infection of NPCs induces their death by multiple mechanisms, including apoptosis, pyroptosis, and autophagy, which contribute to microcephaly in the offspring (60–67).

A critical question is how ZIKV comes into contact with NPCs. Presumably the placenta is to blame as the bridge between the mother and fetus. This transient organ begins to form at implantation and supports the passage of nutrition and gases, toxin elimination, and protection against pathogens (68–70). Injecting ZIKV into the uterus of pregnant animals leads to detectable viral antigens in the placenta, indicating that the virus can infect those cells. Indeed, ZIKV infection of the placenta leads to total loss of boundaries between placental layers, and mixing of maternal and fetal blood (71, 72). Once the virus infects the placenta, it can replicate in cytotrophoblasts and infect macrophages (Hofbauer cells), which then produce IFNα, IL-6, and monocyte chemoattractant protein-1 (MCP-1) (38). Ultimately the virus passes through the placenta and enters the brain.

Once inside the fetal central nervous system, ZIKV can infect NPCs, astrocytes, and microglia, but it is toxic only to NPCs. Microglia, in contrast, allow the virus to replicate but do not die, implying that they can serve as a viral reservoir in the fetal brain (73). Given the developmental origin of the microglia from the yolk sac, the virus likely enters the fetus *via* the maternal vasculature (73, 74). Microglia not only help the virus spread throughout the central nervous system (CNS) (73, 75), but they also induce the secretion of pro-inflammatory cytokines IL-6, IL-1 β, TNF-α, and MCP-1, which contribute to neuroinflammation with long-term consequences (76–78).

Much of what we know about how ZIKV affects the fetal brain comes from studies in cell culture. Given that a Petri dish can be a poor mimic of the diverse, highly structured brain, many researchers have focused on brain organoids (**Figure 2**). Typically, human inducible pluripotent stem cells (iPSC) are used to derive NPCs in a three-dimensional culture system in which specific brain regions can develop. Brain organoids mimic key characteristics of human neurodevelopment, such as forming a complex progenitor zone with abundant outer radial glial cells (79). Interestingly, infecting brain organoids with ZIKV reduces their volume, analogous to microcephaly (61, 80). Such observations have led to the idea that ZIKV targets NPCs through recognition *via* Toll-Like receptor 3, but not neurons (81–83), while downregulating genes related to the cell cycle, cell division, neurogenesis, as well as axonal guidance and differentiation, consistent with the characteristics of microcephalic offspring born to ZIKV-infected mothers (84, 85).

### Cytomegalovirus

This double-stranded DNA virus belongs to the family *Herpesviridae* and genus *Herpesvirus* (86). Congenital CMV infection leads to clinical manifestations in only 10% of newborns, yet 10-15% of apparently asymptomatic newborns have long-term sequelae, such as sensorineural hearing loss, neurodevelopmental disorders, ophthalmic complications, cerebral neoplasms, and ASD (**Figure 2**) (87–89). The virus moves vertically from mother to fetus by infecting cytotrophoblasts in the placenta, where the infection alters placenta integrity and development (90, 91) and dysregulates gene expression to inhibit placental cell differentiation, self-renewal, and migration (39, 92). Similar to ZIKV, CMV infects and replicates in microglia (93). In animal models, CMV infects fetal macrophages, which infiltrate the fetal brain preferentially in the choroid plexus and in ventricular and subventricular areas, where the macrophages induce inflammation by producing IL-6, IL-1β, and TNF-α (94–97).

Once inside the fetal brain, CMV can also directly infect NPCs, likely through platelet-derived growth factor receptor alpha (PDGFRα) (98). Indeed, expressing this receptor in NPC-derived neurospheres *in vitro* allows CMV infection (99). Like ZIKV, CMV also inhibits NPC neurogenesis, proliferation, differentiation and migration (100–104), while stimulating their apoptosis (105). This may help explain why maternal CMV infection is linked to hearing loss, intellectual disability, and other cognitive deficits in offspring (106).

Studies with human brain organoids have provided crucial information about how CMV infection disrupts fetal brain development (**Figure 2**). Exposing brain organoids to CMV alters the structure and organization of the cortex-like component and the distribution of NPCs and neuronal populations in cortical layers (107, 108).

### Influenza A Virus

Influenza A virus is a single-stranded RNA virus belonging to the family *Orthomyxoviridae* and the genus *Alphainfluenzavirus* (109). A seasonal virus, it causes 3-5 million severe cases globally, with pregnant women at particularly high risk of complications following infection. Occasionally, strains of the Influenza virus have caused pandemics that killed millions of people worldwide, such as in 1918 and 2009 (110). Offspring of mothers infected with Influenza A virus show reduced psychomotor development in the first months of life (111), as well as cognitive problems, schizophrenia, and bipolar disorder (**Figure 1B**) (9, 11, 13, 15, 18, 112).

Interestingly, the viral genome has yet to be detected in the placenta or fetal brain (113), suggesting that maternal infection with the virus affects the fetus indirectly by triggering immune responses that compromise neurodevelopment. Increases in maternal levels of IL-6, IL-1β, TNF-α, and IFN-β in response to infection can damage the placenta and intrauterine fetal growth restriction caused by hypoxia that can be detected as upregulation of hypoxic-inducible factor-1α (HIF-1α) in the placenta (114). In pregnant women expressing human leukocyte antigen DRB14, this can induce the production of pro-inflammatory cytokines that cause fetal neuroinflammation, which has been linked to increased risk of schizophrenia in offspring (13). Indeed, postmortem histology of schizophrenic offspring shows disturbed neuronal migration, disorganized lamina strata, ectopic pyramidal cells, abnormal expression of neural cell adhesion molecules, no astrogliosis, as well as reduced densities of axons, dendrites, and synapses. Magnetic resonance imaging shows smaller intracranial volume than in offspring born to uninfected mothers as well as less gray and white matter, especially in the cerebral cortex (115). Animal studies confirm that viral infection of the mother induces immune responses in the fetus that alter critical signaling proteins and pathways in neurodevelopment, such as the fragile X mental retardation protein (FMR1), glutamatergic/GABAergic balance, and reelin signaling (116). Those studies further confirm that viral infection of the mother significantly reduces volumes of the prefrontal, frontal, cingulate, insular, parietal and temporal-auditory regions of the cortex (117) and can lead to impairments in exploratory behavior and social interaction in adulthood (118).

### SARS-CoV-2 

While researchers have explored the effects of maternal infection with ZIKV, CMV and Influenza A virus on fetal neurodevelopment for several years, we are only beginning to examine whether and how the recently emerged SARS-CoV-2 virus, responsible for the current coronavirus disease 2019 (COVID-19) pandemic, may affect newborns in the long term. SARS-CoV-2 infection induces similar cellular responses as the other three viruses, including an increase in pro-inflammatory cytokines associated with disease severity and can be fatal (119). In particular, COVID-19 patients show elevated IL-6 in their serum (120), even prompting the consideration of anti-IL-6 antibodies as a treatment (121, 122). In some patients, peripheral T helper cells also show high levels of interleukin (IL) 17A in the lung (123, 124).

Whether the virus can cross the placenta and infect the fetus is unknown. Several studies have failed to detect viral antigens or RNA in the placenta (125–133), and placental cells do not seem to co- express the two receptors normally required for the virus to enter cells, angiotensin-converting enzyme 2 receptor (ACE2) and transmembrane protease serine 2 (TMPRSS2) (134, 135). On the other hand, others have reported viral infection in the placenta (136–142), but not in the fetal brain, even though ACE2 and TMPRSS2 are expressed in different types of neurons and microglia (143–148) as well as NPCs (145, 149). In fact, NPCs in culture can be artificially infected with SARS-CoV-2 (150, 151). These contradictory findings make it crucial to assess whether SARS-CoV-2 directly infects fetal neural cells. Studies with human iPSCs and brain organoid cultures are essential in this regard, and such work has already described the expression of ACE2 and TMPRSS2 in the brain (152) and choroid plexus (152, 153). Since the choroid plexus is essential for the formation and function of the CNS and cerebrospinal fluid (CSF) (154), SARS-CoV-2 infection of this region leads to the breakdown of the barrier between the blood and CSF, which can allow the passage of virus, immune cells, and cytokines in the brain, where they can promote a pro-inflammatory environment (153) and disrupt the normal choroid plexus function (155). Organoid studies have also shown that SARS-CoV-2 can contribute to neuroinflammation by infecting pericytes and then spreading to neural cells such as astrocytes, in which they induce type I IFN production and ultimately cell death (156).

**Figure 3:**
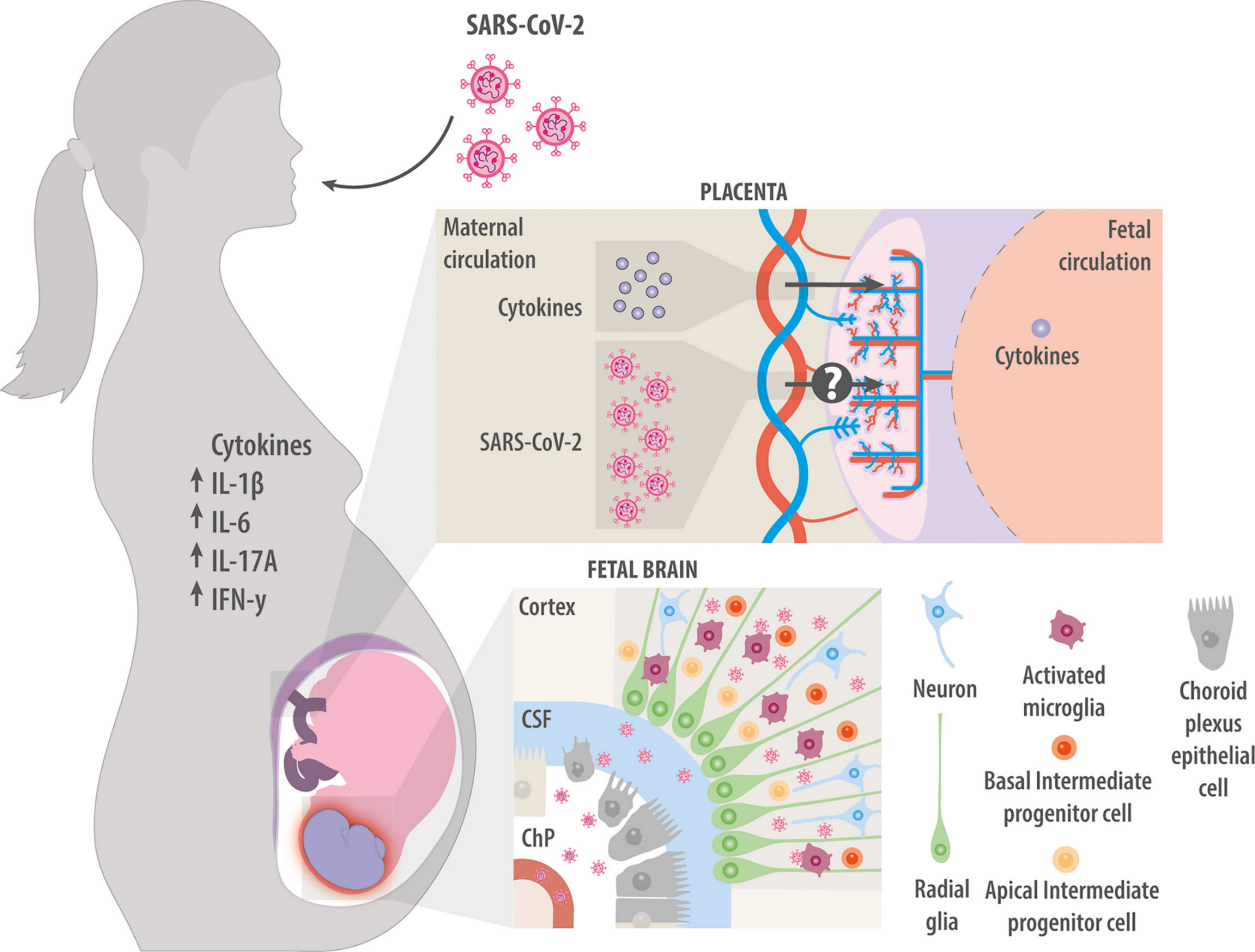
Maternal infection with SARS-CoV-2. Maternal infection with SARS-CoV-2 increases levels of cytokines IL-1β, IL-6, IL-17A, and IFN-γ in the mother. These factors can cross the placenta and induce neuroinflammation in the fetal brain. Whether the virus itself can also cross the placenta and infect the fetus is unclear. Studies *in vitro* suggest that the virus can infect fetal choroid plexus cells, where they disrupt production of cerebrospinal fluid and compromise the integrity of the blood-brain barrier, allowing immune cells and cytokines to enter the developing brain. Studies *in vitro* have also suggested that SARS-CoV-2 can directly infect NPCs and microglia.

Substantial circumstantial evidence points to the ability of SARS-CoV-2 to directly affect the developing brain, but much more remains to be clarified, especially with respect to viral effects on NPCs and cortical development. For this work, well-designed studies are needed with cortical organoids and assembloids involving the various actors involved in neurodevelopment, such as immune system cells, choroid plexus and microglia (**Figure 3**).

Ultimately, appropriate animal models are needed, which has been a challenge so far because the viral “spike” protein binds only weakly to the ACE2 receptor in mice (157). Researchers can use the transgenic mouse “B6. Cg-Tg(K18-ACE2)2Prlmn/J”, which was developed during the SARS-CoV epidemic in 2003 and which expresses the human ACE2 receptor (158). The virus can infect epithelial cells in the upper and lower respiratory tract and brain of this mouse. In addition, the “mouse-adapted” recombinant SARS-CoV-2 virus called “SARS-CoV-2 DA” can enter mouse cells (159). These two models will likely provide important insights into how maternal infection with SARS-CoV-2 affects offspring.

Analysis of brain organoids and assembloids may be powerful for elucidating the effects of not only SARS-CoV-2, but also better studied viruses like ZIKV and CMV, on fetal neurodevelopment. For example, combining the use of assembloids and single-cell analysis may clarify how viruses influence the interactions among NPC populations, newborn neurons and microglia in different brain areas. Such work may help identify how viral infection alters gene expression and signaling pathways in NPCs and microglia, and determine whether viral effects on offspring depend on the gestational age at infection. Age-dependent effects may help explain why ZIKV and CMV target the same cells, yet exert different long-term effects on the offspring. Ultimately, such studies may lead to strategies that prevent viral entry and replication in fetal cells, and reduce the production of pro-inflammatory cytokines that can fight the infection and wreak havoc on the developing fetal brain.

## Pro-Inflammatory Cytokines And Fetal Neurodevelopment

The release of pro-inflammatory cytokines by maternal immune cells in response to infections can reach the developing fetus through the placenta (160). Furthermore, considering that the primordial fetal blood-brain barrier (BBB) is highly permeable throughout development, the maternal pro-inflammatory cytokines can enter the fetal circulation and profoundly effects the fetal brain (161). This likely explains why elevated levels of pro-inflammatory cytokines in the fetal brain and cerebrospinal fluid have been associated with neuroinflammation and neurodevelopmental diseases (162–166).

### IL-6

IL-6 has well-established effects on neurodevelopment. It is produced by various cell types, including fibroblasts, endothelial cells, and immune cells. In the innate immune system, myeloid cells such as neutrophils, monocytes/macrophages, and dendritic cells recognize pathogens through Toll-like receptors, then produce IL-6 (167). Higher levels of maternal IL-6 during pregnancy have been linked to lower cognitive ability in offspring during the first year of life, suggesting an altered front limbic circuit (168). Higher levels of maternal IL-6 during pregnancy have also been linked to an enlarged right amygdala and greater bilateral connectivity of the amygdala to brain regions involved in sensory processing and integration (spindle-shaped somatosensory cortex), salience deletion (insula anterior), and learning and memory (caudate and parahippocampal gyrus) in 2-year-old offspring (169). These findings may reflect the effects on IL-6 on working memory, cognitive function and behavior (169).

Studies in animal models have begun to clarify how IL-6 exerts its effects on fetal neurodevelopment. IL-6 readily crosses the fetal BBB (170, 171). Intraperitoneal administration of IL-6 to pregnant females results in inflammation of the fetal brain and causes social, motor, and learning deficits in the offspring as adults (**Figure 2**) (172, 173). Intraventricular injection of IL-6 on postnatal day 0.5 increases the formation of excitatory synapses while reducing inhibitory synapses, cognitive and learning functions, and social interactions (174). Transgenic mice that overexpress IL-6 in the brain show reduced neurogenesis in the dentate gyrus of the hippocampus (175). These perturbations are linked to an expansion of NPCs in the adult forebrain and to altered proportions of interneuron subtypes during olfactory neurogenesis in the offspring as adults. Moreover, IL-6 levels in the embryo regulate the size of the NPC pool, and the burst of maternal IL-6 expression upon infection permanently increases this pool, which persists into adulthood (176, 177). Elevated maternal IL-6 may also delay the migration of progenitor cells of GABAergic neurons from the ventral telencephalon to the developing cortical plate (178).

Infection of pregnant mice causes an increase in IL-6 in both maternal serum and fetal brain, and loss of Purkinje cells in lobe VII of the cerebellum. These changes are associated with phosphorylation of signal transducer and activator of transcription (STAT) 3, a regulator of genes related to cell proliferation and apoptosis, in the fetal hindbrain. In support of the role of IL-6 in these brain alterations, ablating the receptor for IL-6 from placental trophoblasts mitigates or even prevents the alterations as well as the accompanying autistic-like behaviors (179). In addition to this receptor in the placenta, microglia help mediate the effects of IL-6: the cytokine increases the branching and vacuole density of microglia, and activated microglia themselves produce IL-6, exacerbating its adverse effects during neurodevelopment (180).

### IL-17A

IL-6 and other cytokines help drive the transition from the initial innate immune response to the specific adaptive immune response led by T cells (181). IL-6 in combination with TGF-β promotes the differentiation of naive T cells towards helper T cells with a Th17 phenotype (182). Th17 cells produce IL-17A (183) in response to fungal, bacterial and viral infections (184–186). Infection of pregnant mice has been linked to alterations in IL-17A, which in turn lead to fetal neurodevelopmental problems (187–189). Administering IL-17A to the mother or directly into the embryonic brain activates microglia in the subventricular zone and increases their phagocytic ability (**Figure 2**) (180), leading to a smaller cortex in the embryo and in the offspring as adults. IL-17A also upregulates gene expression at GABAergic synapses and reduces anxiety-like behavior in offspring (190).

IL-17A binds to NPCs *via* a specific surface receptor, inhibiting their proliferation as well as their ability to form neurospheres or to differentiate into astrocytes and oligodendrocyte precursor cells (35). In addition, IL-17A acts to disorganize cytoarchitecture in the cortex, reflected in delayed expression of neuronal markers in different cortical layers (191). These cortical alterations are associated with ASD-like behaviors in the offspring, including abnormal ultrasonic vocalization, deficits in social interaction, and repetitive behavior. The tight link between maternal cytokine signaling and fetal neurodevelopment is reflected in the observation that maternal infection upregulates the IL-17 receptor in the fetal cortex, but not if the mother is pretreated with an anti-IL-17A antibody (191).

### IL-1β

During an infection, IL-1β is one of the first cytokines released by macrophages, monocytes, and dendritic cells. It is required for efficient innate and adaptive immune responses (192). Intraperitoneal injection of IL-1β into pregnant animals (**Figure 2**) increases the density of amoeboid microglia in the cortical plate, suggesting inflammation of the fetal brain (193). In addition, IL-1β injection into pregnant female induces CD4+ and CD8+ T cells to infiltrate the placenta and ultimately enter the fetus, where they thin out the cerebral cortex and cause behavioral deficits later in the offspring (194). Decreasing IL-1β expression in the placenta and fetal brain as well as inhibiting the enzyme nitric oxide synthase in the cortex reverse the ability of IL-1β to disorganize the arborization of neuronal dendrites in the offspring (195). The harmful effects of IL-1β and IL-6 on fetal brain can also be reversed by intraperitoneal administration of an interleukin-1 receptor antagonist into pregnant animals (196): the antagonist reduces damage to the placenta, and in the offspring, it reduces the density of microglia expressing ionized calcium-binding adaptor molecule 1 (Iba-1) and improves motor performance.

### IFN-γ

IFN-γ is secreted by natural killer cells as part of the innate immune response and by CD4+ Th1 cells and CD8+ T cells as part of the adaptive immune response. In mice, IFN-γ inhibits NPC proliferation by blocking them in G1/S through a pathway that involves STAT1 (197). Injecting IFN-γ into the fetal brain ventricle on embryonic day 9.5 leads to a smaller number of immature neurons and larger number of nestin-positive NPCs on embryonic day 14.5, suggesting that IFN-γ inhibits embryonic neurogenesis (198). These effects can be reversed by inhibiting signaling downstream of the IFN-γ receptor or by knocking down STAT1 results in a reversal of the adverse effects of IFN-γ administration on neurogenesis (198).

In these ways, the maternal inflammatory response to viral infection induces pro-inflammatory cytokines that can reach the developing fetal brain *via* the placenta and the fetal BBB. These cytokines exert their effects on NPCs and in microglia leading to their activation and long-lasting neuroinflammation. These “indirect” effects of maternal infection add to the direct effects caused by vertical virus transmission, emphasizing the importance of developing vaccines against viruses that affect fetal neurodevelopment and encouraging vaccination among pregnant women or trying to become pregnant. These considerations also highlight the need to develop comprehensive anti-viral strategies that neutralize the virus and dampen the strong maternal immune response that can harm fetal neurodevelopment.

## Discussion

Maternal infections during pregnancy can have severe effects on fetal brain development, causing long-term consequences manifesting as ASD, schizophrenia, social disturbances, cognitive impairment, and other disorders. This review has discussed two of the major mechanisms by which a maternal infection can alter neurodevelopment. First, vertical transmission of virus between mother and fetus *via* the placenta allows the virus to enter the fetal circulation, from where it infects cells important for neurogenesis, such as NPCs and microglia. Second, viral infection in the mother triggers an increase in the levels of pro-inflammatory cytokines in her systemic circulation, which then enter the fetal circulation and ultimately reach the developing brain. These cytokines alter NPC biology and activate microglia, causing them to produce even more pro-inflammatory cytokines that maintain neuroinflammation. Whether fetal brain cells are directly infected by virus or affected by cytokines coming from other infected cells, the fetal cells undergo various changes in response to maternal infection, leading to altered cortical structure, brain size, or connectivity between brain areas. These perturbations can ultimately affect the cognitive ability and behavior of the offspring into adulthood.

The severe SARS-CoV-2 pandemic has brought new urgency to research into how maternal infection with virus can affect fetal neurodevelopment. It is imperative that we understand whether SARS-CoV-2 can pass from mother to fetus, and whether it can directly infect fetal neural cells and microglia. However, as this review illustrates, the virus may affect fetal neurodevelopment even without directly infecting it, so studies should examine whether maternal infection with SARS-CoV2 is associated with neurodevelopmental disorders. Such studies should examine COVID-19 patients but also exploit the experimental tools of human iPSC-derived brain organoids and assembloids, which have proven so powerful in research on ZIKV and CMV. Such studies should also make use of appropriate mouse models. The overarching goal is to prevent or safely manage viral infections during pregnancy and minimize their long-term consequences on the offspring.

## Author Contributions

DE and GC drafted the manuscript, which all authors revised. All authors contributed to the article and approved the submitted version.

## Funding

DE is supported by a Postdoctoral Grant from ANID FONDECYT (3200846), and GC by a Returnee Fellowship from IBRO. This work was funded by Regular Grants from ANID FONDECYT (1161374, 1210507), a REDES Grant from ANID PCI (180113), and a Start-Up Grant from Universidad Mayor. PM is supported by ANID FONDECYT Iniciación (11190258) ER is supported by an ANID FONDECYT Regular 1191526 grant.

## Conflict of Interest

The authors declare that the research was conducted in the absence of any commercial or financial relationships that could be construed as a potential conflict of interest.

## Publisher’s Note

All claims expressed in this article are solely those of the authors and do not necessarily represent those of their affiliated organizations, or those of the publisher, the editors and the reviewers. Any product that may be evaluated in this article, or claim that may be made by its manufacturer, is not guaranteed or endorsed by the publisher.

## Abbreviations

ASD, autism spectrum disorder; BBB, blood-brain-barrier; CDn, cluster of differentiation CMV, Cytomegalovirus; CNS, central nervous system; COVID-19, coronavirus disease 2019; CSF, cerebral spinal fluid; IFNn, interferon; IL-1β, interleukin 1 beta; IL-6, interleukin 6; IL-17A, interleukin 17A; iPSC, inducible pluripotent stem cells, NPCs, neural stem/precursor cells; SARS-CoV-2, severe acute respiratory syndrome coronavirus 2; THn, T helper; TMPRSS2, transmembrane protease serine 2; TNF-α, tumor necrosis factor alpha; ZIKV, Zika virus.
